# LungSurg: A Generative AI System for Segmentation and Phase Classification in Thoracoscopic Lobectomy

**DOI:** 10.1002/mco2.70613

**Published:** 2026-01-20

**Authors:** Hengrui Liang, Zeping Yan, Yudong Zhang, Keyao Dai, Hongyan Li, Jianfei Shen, Pengfei Li, Jipeng Jiang, Guochao Zhang, Xiang Zhang, Hao Chen, Honglang Zhang, Yuzhuo Zhang, Shujun Liang, Minsheng Chen, Xin Wang, Anyi Rao, Wei Wang, Lei Zhao, Yuchen Guo, Jianxing He

**Affiliations:** ^1^ Department of Thoracic Surgery, China State Key Laboratory of Respiratory Disease and National Clinical Research Center for Respiratory Disease The First Affiliated Hospital of Guangzhou Medical University Guangzhou China; ^2^ Department of Thoracic Surgery Zhongda Hospital Southeast University Nanjing China; ^3^ Department of Thoracic Surgery Taizhou Hospital of Zhejiang Province Affiliated to Wenzhou Medical University Linhai China; ^4^ Department of Thoracic Surgery Cancer Hospital of Dalian University of Technology Cancer Hospital of China Medical University Liaoning Cancer Hospital and Institute Shenyang Liaoning China; ^5^ Department of Thoracic Surgery First Medical Center of Chinese People's Liberation Army General Hospital Beijing China; ^6^ Department of Thoracic Surgery National Cancer Center/National Clinical Research Center for Cancer/Cancer Hospital Chinese Academy of Medical Sciences and Peking Union Medical College Beijing China; ^7^ Department of Thoracic Surgery The First Affiliated Hospital of Wenzhou Medical University Wenzhou Zhejiang China; ^8^ Department of Thoracic Surgery Key Laboratory of Cardio‐Thoracic Surgery (Fujian Medical University), Fujian Province University, Clinical Research Center for Thoracic Tumors of Fujian Province, National Key Clinical Specialty of Thoracic Surgery Fuzhou Fuzhou China; ^9^ Clinical Medicine Guangzhou Medical University Guangzhou China; ^10^ Department of Pancreatic Surgery West China Hospital Sichuan University Chengdu China; ^11^ Department of Information Engineering The Chinese University of Hong Kong Hong Kong China; ^12^ School of Basic Medical Sciences Guangzhou Medical University Guangzhou China; ^13^ Institute for Brain and Cognitive Sciences Beijing National Research Center for Information Science and Technology (BNRist) Tsinghua University Beijing China

**Keywords:** deep learning, generative artificial intelligence, lobectomy, surgical education, thoracic surgery

## Abstract

The integration of artificial intelligence (AI) into surgical practices is advancing towards greater intelligence and precision. This study assesses the potential of AI in video‐assisted thoracoscopic surgery (VATS) lobectomy for lung cancer by developing an AI system named LungSurg. LungSurg comprises two interconnected networks: a segmentation network for identifying intrathoracic anatomy and surgical instruments, and a classification network for recognizing surgical phases. We prospectively collected 222 VATS lobectomy videos from eight centers, generating over 32,000 annotations and more than one million frames with phase information. In external validation, the segmentation network achieved mean Average precision scores of 0.745 for the left lung and 0.726 for the right lung across various instruments and anatomical structures. The classification network demonstrated Top‐1 and Top‐3 accuracies of 71.5% and 88.0%, respectively, in identifying 14 surgical phases. Comparative experiments revealed that LungSurg performed comparably to senior surgeons in anatomical identification and surpassed them in sensitivity. In addition, an educational study showed that surgical residents trained with LungSurg significantly improved their anatomical identification and phase classification skills compared to those using conventional methods. These results indicate that LungSurg accurately analyzes VATS lobectomy procedures, highlighting the feasibility and potential of AI‐driven tools in enhancing thoracic surgical practices.

## Graphical abstract

The study introduces LungSurg, an AI system designed to assist in video‐assisted thoracoscopic (VATS) lobectomy for lung cancer by accurately identifying anatomical structures and surgical phases. The system outperforms senior surgeons in anatomical sensitivity and improves surgical residents' skills in phase classification and anatomical identification.

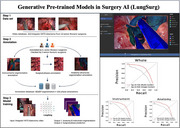

## 1 Introduction

Lung cancer remains the leading cause of cancer‐related mortality worldwide, with approximately 2.2 million new cases and 1.8 million deaths annually [1]. For early‐stage non‐small cell lung cancer (NSCLC), video‐assisted thoracoscopic surgery (VATS) lobectomy with systematic lymph node dissection is the standard treatment, offering oncological outcomes comparable to open thoracotomy but with benefits such as reduced postoperative pain, shorter hospital stays, and faster recovery [2, 3].

Mastering VATS lobectomy poses considerable challenges due to its technical complexity and the intricate anatomy of the thoracic cavity. Surgeons require a substantial number of cases to achieve proficiency, highlighting a steep learning curve [4]. For surgical residents, the initial phase of training involves acquiring comprehensive anatomical knowledge and understanding the sequential steps of the operation [5]. However, limited access to real‐time mentorship and detailed educational resources often hampers this learning process, underscoring the need for innovative training tools [6].

Artificial intelligence (AI) has emerged as a promising solution to enhance surgical training and intraoperative performance [7]. In less complex procedures like laparoscopic cholecystectomy and cataract surgery, video‐based AI systems have successfully segmented anatomy, identified instruments, and classified procedural steps, contributing to improved surgical outcomes [8, 9, 10]. Our team has previously introduced innovative AI solutions: SurgSmart, an AI system for quality control [11]; Surgesture, offering objective skill assessment through novel instrumentation [12]; and algorithms for objective skill classification based on surgical gestures [13]. However, applying AI to complex procedures like VATS lobectomy remains underexplored due to high anatomical variability and intraoperative factors such as occlusion by instruments, smoke, and tissue deformation [14].

Advanced techniques like image inpainting–which uses generative learning to reconstruct missing or occluded image parts–can enhance AI's interpretation of complex surgical fields [15]. In thoracic surgery, this approach restores visual continuity when key structures are obscured, potentially improving segmentation and phase classification in VATS lobectomy.

In this study, we developed *LungSurg*, an AI system tailored for VATS lobectomy. LungSurg integrates image inpainting to address occlusion and anatomical variability, enabling precise segmentation of anatomical structures and surgical instruments, as well as accurate procedural phase classification throughout the operation. We validated LungSurg in a multicenter cohort, demonstrating its feasibility and effectiveness. By applying advanced generative AI techniques, our work aims to lay the foundation for future applications in automated quality assessment and enhanced surgical training, which may ultimately contribute to improved surgical practice.

## 2 Results

### 2.1 Video Characteristics and Annotations

**FIGURE 1:**
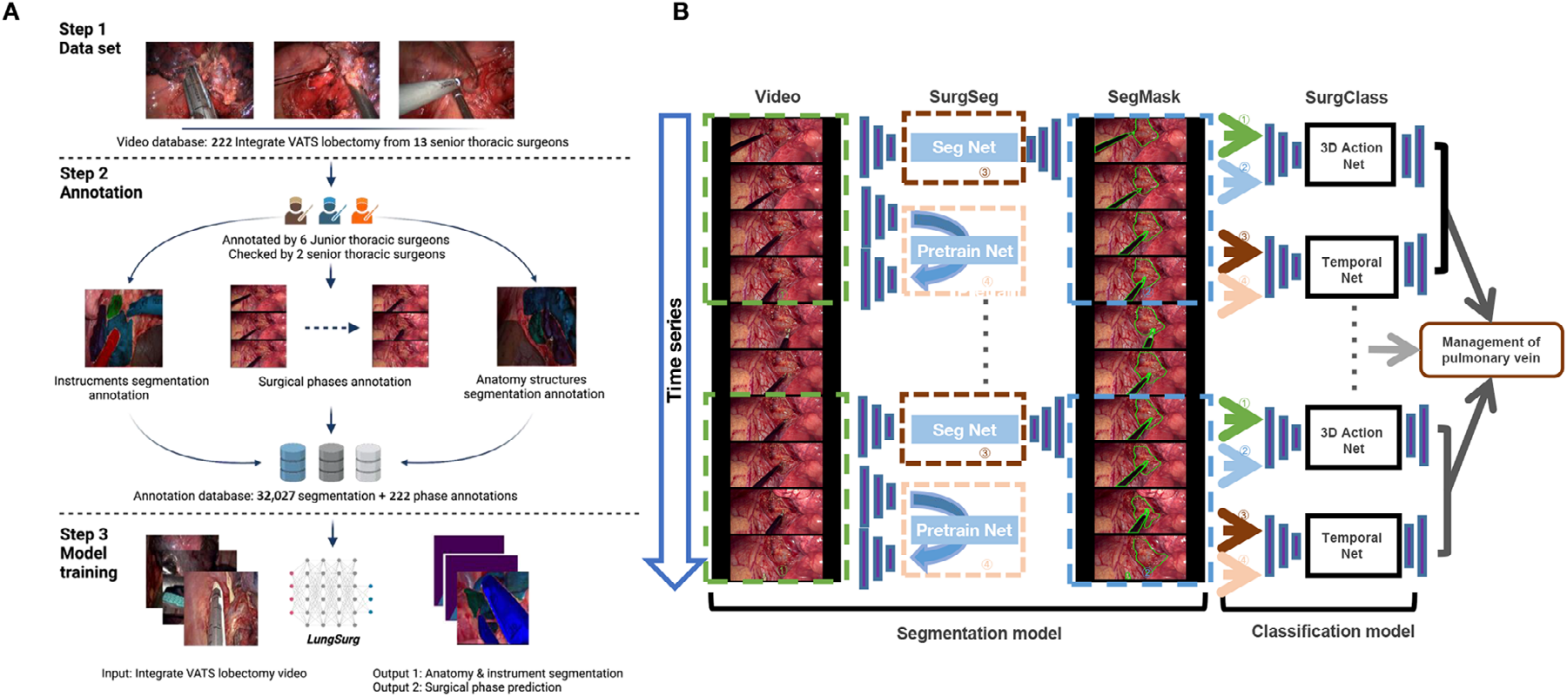
The study design and the establishment of the artificial intelligence model. (A) The workflow and data requirements for the *LungSurg*. (B) The whole system architecture of the *LungSurg*. ①*SurgSeg*’s judgment confidence for each pixel of the image; ② the result of the dot product between the original image and the segmentation mask given by *SurgSeg*; ③ image segmentation features generated by the encoder of *SurgSeg*; ④ image features generated by the encoder of the pre‐trained network.

In this multicenter study, involving eight centers, we analyzed a total of 222 VATS lobectomy videos encompassing complete surgical procedures–from endoscope insertion to wound closure (Figure 1A). These videos included all lung lobes (right upper lobe [RUL], right middle lobe [RML], right lower lobe [RLL], left upper lobe [LUL], and left lower lobe [LLL]) and were performed by senior thoracic surgeons, ensuring a comprehensive representation of surgical practices across different institutions.

**FIGURE 2:**
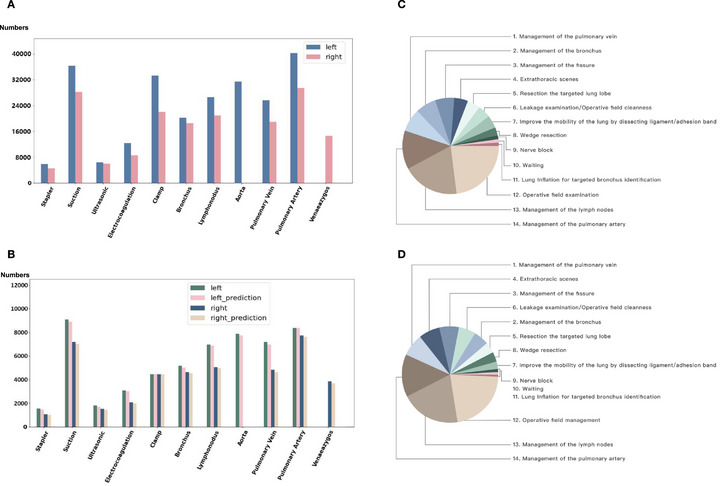
The representative annotations and predictions of all labels in different surgical structures.The distribution of the annotated segmentation and the proportion of each category in surgical phase. (A) Annotation in training set of segmentation; B: Annotation and prediction in validation set of segmentation. (C) Annotation in training set of phase Classification; D: Prediction in validation set of phase Classification.

The dataset was divided into a training set comprising 165 videos (Table S1a) from four centers and a validation set of 57 videos (Table S1b). After frame extraction at a rate of one frame per second (fps), approximately 1.5 million frames were generated for analysis. A total of 32,027 surgical structures were manually annotated, averaging about 144 annotations per video. The distribution of annotations was consistent between the training and validation sets, as well as predicted labels, providing a diverse and balanced dataset for model development (Figure 2A,B).

All frames were labeled with corresponding phase information, and a total of 14 definitive surgical phases were identified (Table S1, Figure S1). The duration of each phase varied due to differences in surgical techniques and patient anatomy. Core surgical steps, such as the management of bronchovascular structures, lymphadenectomy, fissure dissection, and lobe resection, accounted for the largest proportion of surgical time (Figure 2C,D).

### 2.2 Interobserver Agreement on Annotations

To ensure high‐quality and consistent annotations, we employed a double‐check method complemented by validation from a third expert. Six certified thoracic surgeons were divided into two groups, with videos randomly allocated in a 1:1 ratio. Within each group, surgeons collaborated to reach consensus on annotations for each video. The annotation consistency in the segmentation task was assessed using metrics such as positive identification rate (PID), positive identification confidence (PIC), and sensitivity identification rate (SID), yielding values of 0.952, 0.992, and 0.971, respectively. For the surgical phase classification task, the frame‐level agreement Cohen's kappa score was 0.948 overall, with 0.942 for right lobectomy and 0.951 for left lobectomy. These high agreement scores indicate excellent consistency in annotation quality across centers.

### 2.3 Performance of LungSurg on the Segmentation Task

**FIGURE 3:**
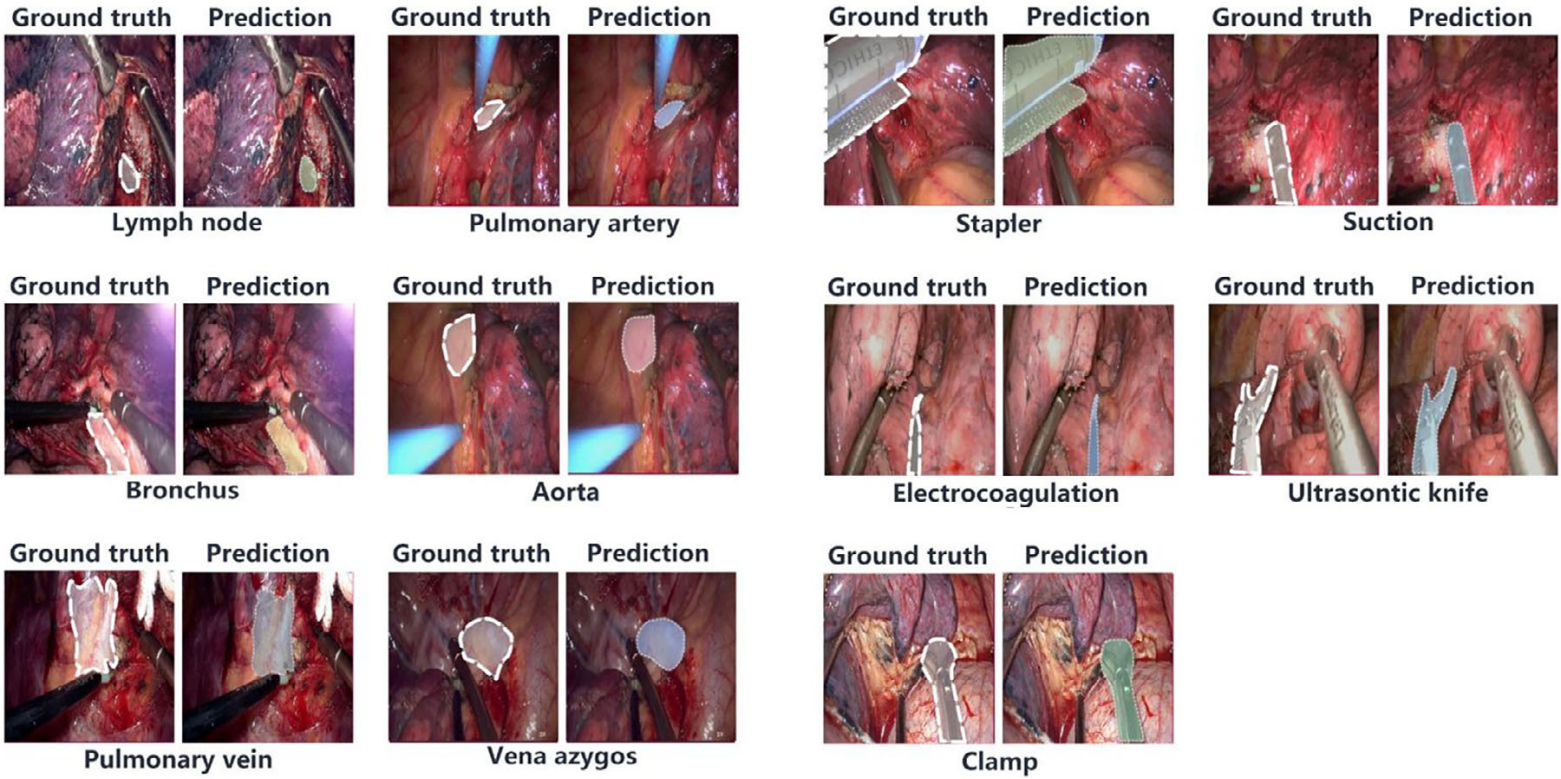
The representative annotations and predictions of all labels in different surgical structures.

The distribution of predicted labels closely matched the annotated ground truth in the validation set (Figure 2B). Visual comparisons of the model's predictions with manual annotations confirmed the high segmentation performance (Figure 3).

In the external validation set from three centers, the segmentation network (*SurgSeg*) of LungSurg demonstrated robust performance across all structure categories. The mean average precision (mAP) at an intersection over union (IoU) threshold of 0.5 was 0.745 for the left lung and 0.726 for the right lung for all structures (Table 1). Specifically, the mAP of anatomical structures was 0.670 for the left lung and 0.679 for the right lung. Regarding surgical instruments, the mAPs were 0.806 for the left lung and 0.755 for the right lung (Table 1). Major anatomical structures, such as pulmonary vessels and bronchi, achieved mAP values ranging from 0.575 to 0.777, indicating accurate segmentation despite anatomical variability and occlusions (Table 1).

**TABLE 1:** The overall segmentation performance of the *LungSurg* in internal and external validation sets.

	Internal validation		External validation	
Classes	Left	Right	Left	Right
Overall	0.816	0.780	0.745	0.726
Overall instruments	0.937	0.902	0.806	0.755
Electrocoagulation	0.972	0.973	0.864	0.797
Ultrasonic knife	0.948	0.950	0.777	0.719
Stapler	0.909	0.833	0.868	0.762
Suction	0.955	0.921	0.788	0.756
Clamp	0.903	0.838	0.700	0.688
Overall anatomy	0.724	0.691	0.670	0.679
Common features				
Pulmonary artery (PA)	0.710	0.653	0.607	0.615
Pulmonary vein (PV)	0.742	0.678	0.601	0.732
Bronchus (B)	0.615	0.671	0.667	0.575
Lymph node (LN)	0.694	0.645	0.777	0.751
Right side				
Vena azygos	—	0.810	—	0.693
Left side				
aorta	0.858	—	0.723	—

*Note*: The indicator was the mean average precision.

**FIGURE 4:**
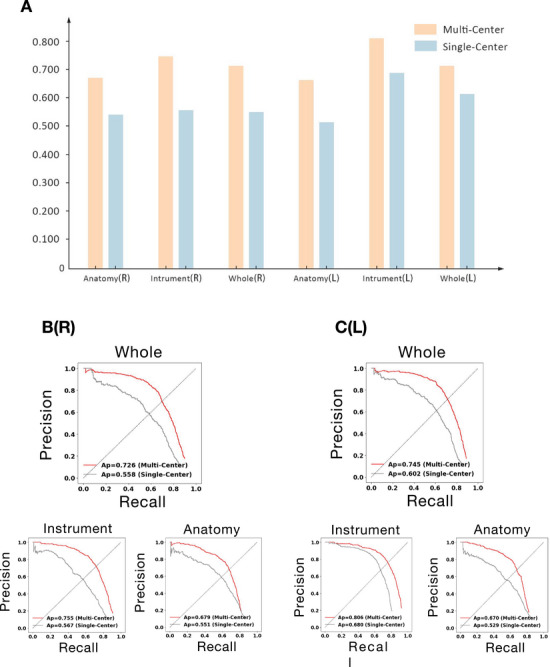
The segmentation performance of the LungSurg in the overall, the left and the right Video‐assisted Thoracoscopic Lobectomy in the external validation set. (A) mAP bar chart for single‐center vs. multi‐center trained models on external validation set. (B) PRC (whole, instrument, anatomy) for SurgSeg (right lung) results. (C) PRC (whole, instrument, anatomy) for SurgSeg (left lung) results. The “R” refers to the right pulmonary lobectomy, and the “L” indicates the left side. mAP, mean Average Precision; PRC, Precision‐Recall Curve.

Figure 4A and Table S2 demonstrate that the model trained on the multi‐center dataset outperforms the single‐center model in both anatomical and instrument segmentation (mAP). Figure 4B further indicates that the multi‐center trained model significantly outperforms the single‐center model. Figure S2 and Table S3 illustrate the model's segmentation performance on surgical anatomy and instruments across three different external validation centers, demonstrating a high degree of visual consistency.

To evaluate the impact of algorithmic improvements, we conducted an ablation study (Table S4) and plotted PRCs for 10‐fold cross‐validation (Figure S3). The baseline ResNet‐50 model attained mAP values of 0.766 on the left and 0.672 on the right. Integrating the Feature Pyramid Network (FPN) improved these results to 0.768 and 0.685, respectively, while further adding non‐local (NL) modules raised them to 0.776 and 0.724, and subsequently incorporating multi‐head self‐attention (MHSA) achieved 0.789 and 0.745, highlighting the effectiveness of these architectures in enhancing the backbone structure. Building upon these improvements, applying Masked Autoencoders (MAE) pre‐training and introducing a co‐occurrence loss function yielded final mAP scores of 0.816 on the left and 0.780 on the right, demonstrating the cumulative benefits of these proposed techniques in enhancing detection performance.

### 2.4 Performance of LungSurg on the Classification Task

For the surgical phase classification task, the classification network (*SurgClass*) achieved a Top‐1 accuracy of 0.715 and a Top‐3 accuracy of 0.880 on the external validation set (Table 2). The distribution of predicted phases closely mirrored the annotations (Figure 2C,D), demonstrating the model's ability to generalize across different surgical styles and patient anatomies.

**TABLE 2:** The overall classification performance of the *LungSurg* in internal and external validation sets.

	Internal validation		External validation	
	Top 1	Top 3	Top 1	Top 3
Overall	0.760	0.929	0.715	0.880
Wedge resection	0.642	0.900	0.625	0.910
Mobilize the lung by dissecting ligament/adhesion band	0.701	0.902	0.700	0.856
Management of the pulmonary vein	0.894	0.993	0.796	0.988
Management of the fissure	0.745	0.954	0.770	0.950
Management of the pulmonary artery	0.791	0.998	0.785	0.804
Management of the lymph nodes	0.822	0.983	0.702	0.964
Operative field management	0.932	0.995	0.909	0.995
Management of the bronchus	0.872	0.951	0.678	0.810
Resection the targeted lung lobe	0.681	0.933	0.763	0.879
Leakage examination/operative field cleanness	0.737	0.897	0.785	0.900
Nerve block	0.608	0.902	0.539	0.870
Extrathoracic scenes	0.903	0.939	0.755	0.848
Waiting	0.671	0.776	0.661	0.803
Lung Inflation for targeted bronchus identification	0.646	0.874	0.544	0.737

*Note*: The indicator (Top1 and Top 3) was the accuracy.

Notably, key procedural steps involving bronchovascular management–such as the handling of the pulmonary artery (PA), pulmonary vein (PV), bronchus, and lymph nodes (LNs)–achieved high Top‐1 accuracies ranging from 0.678 to 0.796 and Top‐3 accuracies from 0.804 to 0.988. These results indicate the model's proficiency in recognizing critical phases essential for surgical success and patient safety. Table S5 demonstrates that the model trained on the multi‐center dataset outperforms the single‐center model in both Top 1 and Top 3 classification performance.

**FIGURE 5:**
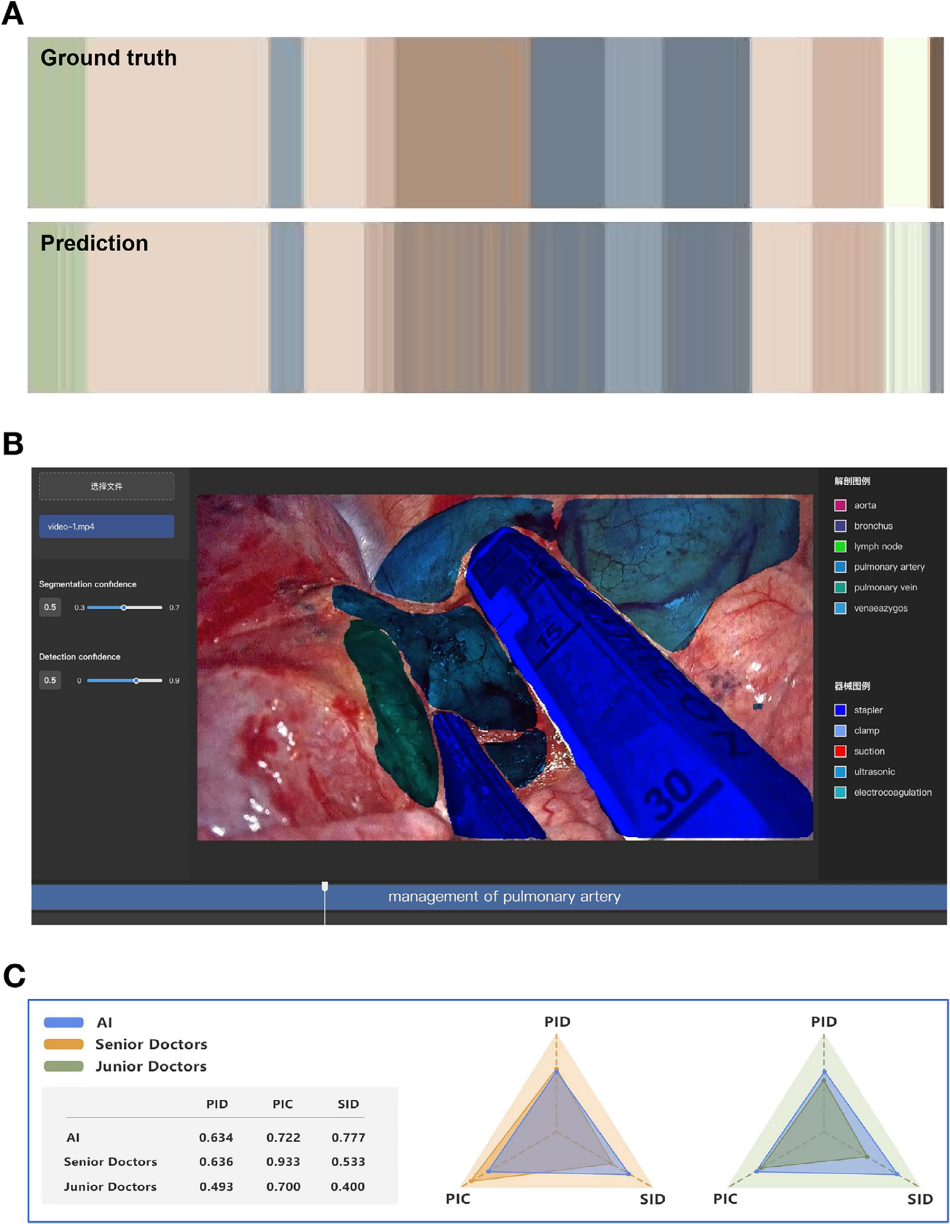
Visualization and expert‐AI comparison of the LungSurg Model. (A) Phase Classification Result Compared to Ground Truth in a Color‐Coded Ribbon Illustration. The Horizontal Axis Represents Time Progression During Surgery. The Upper Ribbon is the Ground Truth, and the Lower is the Predicted Phase. Different Colors Represent Different Phases Corresponding to Figure 2C and 2D. (B) Designed Software Showing Segmentation and Phase Classification Predicted by LungSurg in Real Surgery. The colored outlines represent the contours of the segmentation masks generated by SurgSeg. (C) Expert‐ LungSurg comparison. AI, Artificial Intelligence; PIC, Precision of Instance Classification; PID, Precision of Instance Detection; SID, sensitivity of instance detection.

To visualize the model's accuracy, we compared the phase classification results with the ground truth for a representative case using a color‐coded ribbon illustration (Figure 5A). The nearly complete agreement between predicted and actual phases underscores the model's effectiveness. Furthermore, we integrated the LungSurg model into a custom‐designed software platform (Figure 5B; Demos S1 and S2), enabling automatic extraction of surgical content and generation of predictive images and phase names with segmentation masks, facilitating practical application in clinical settings.

An ablation study of the classification model's performance after algorithmic improvements was conducted (Table S6). Transitioning from local mode analysis (8‐s clips) to global mode analysis (entire videos) improved the Top‐1 and Top‐3 accuracies by 8.6% and 3.9%, respectively. Incorporating segmentation masks (SegMask) into the action network increased the overall global Top‐1 and Top‐3 accuracies by 6.1% and 11.3%, respectively. Adding segmentation features to the temporal network further enhanced the classification performance by 1.9% and 6.3%. The final *SurgClass* model, combining the best features of the action network and temporal network, achieved the highest global Top‐1 and Top‐3 accuracies of 0.757 and 0.929, respectively.

### 2.5 Comparison of LungSurg With Human Experts

To assess LungSurg's performance relative to human expertise, we conducted a comparison experiment using randomly selected short video segments (1–3 s) from the validation set. Three senior surgeons and five junior surgeons participated, annotating vital anatomical structures such as arteries, veins, and bronchi. Performance metrics were calculated by averaging within each group.

In terms of the PID, LungSurg's detection rate was comparable to that of senior surgeons (PID = 0.634 vs. 0.636). For the PIC, senior surgeons achieved a score of 0.933, while LungSurg scored 0.722, similar to junior surgeons (0.700). This suggests that senior surgeons were more confident in their annotations, possibly due to experience. However, LungSurg exhibited a higher SID of 0.777, outperforming senior surgeons by 0.244, indicating greater sensitivity in detecting anatomical structures (Figure 5C). These findings demonstrate that LungSurg performs at a level comparable to experienced surgeons in anatomical identification.

### 2.6 LungSurg‐Assisted Surgical Education

In our pilot randomized study, the experimental group (*n* = 10) trained with LungSurg demonstrated markedly superior performance across all evaluated tasks compared to the control group (*n* = 10) using conventional methods.

For the anatomical structure identification task, the experimental group achieved a mean accuracy of 73.40% (95% CI, 69.5%–77.3%), significantly higher than the control group's 63.0% (95% CI, 58.8%–67.2%; *χ* ^2^ = 13.58, *p* < 0.001). Similarly, in surgical instrument identification, the experimental group's accuracy (88.20%; 95% CI, 85.4%–91.0%) was significantly greater than that of the control group (79.60%; 95% CI, 76.1%–83.1%; *χ* ^2^ = 13.68, *p* < 0.001). An improvement was also observed in the surgical phase classification task, where the experimental group achieved an accuracy of 79.2% (95% CI, 75.6%–82.8%), and an increase over the control group's 65.4% (95% CI, 61.2%–69.6%; 23.77, *p* < 0.001).

## 3 Discussion

In this multicenter study, we developed and validated *LungSurg*, an advanced AI system designed to assist in VATS lobectomy. Our findings demonstrate that LungSurg can accurately identify critical anatomical structures and surgical instruments, as well as classify procedural phases. This work represents a significant advancement in the integration of AI into complex surgical procedures, with far‐reaching implications for surgical education, intraoperative assistance, patient outcomes, and the broader landscape of surgical technology.

### 3.1 Advancements in Surgical AI for VATS Lobectomy

While previous AI applications in surgery have predominantly targeted less complex and more standardized procedures such as laparoscopic cholecystectomy and cataract surgery [16, 17], our work extends this frontier to VATS lobectomy, a domain presenting a distinct set of formidable challenges. Unlike cholecystectomy, which has a relatively predictable anatomy and procedural sequence, VATS lobectomy is characterized by significant inter‐patient anatomical variability, a highly dynamic and non‐rigid surgical field due to lung motion, and the critical need to identify and preserve delicate bronchovascular structures that are often obscured by tissue, smoke, or instruments.

Recent advancements in AI‐driven surgical assistance, such as the integration of deep learning models for decision support, have shown promise in enhancing surgical precision and reducing operative times [18]. LungSurg contributes to this growing body of work by not only segmenting anatomical and instrumental elements but also by accurately classifying surgical phases, thereby providing a comprehensive tool for intraoperative guidance.

To our knowledge, LungSurg is the first comprehensive AI system to successfully tackle both anatomical segmentation and phase classification in this high‐stakes environment. Previous systems like Surgesture [13] and SurgSmart [11] laid crucial groundwork in abdominal surgery, but their architectures were not designed to handle the unique visual and contextual complexities of thoracic surgery. By demonstrating expert‐level performance in VATS lobectomy, our study validates the feasibility of deploying advanced AI in one of the most technically demanding areas of minimally invasive surgery, thereby opening a new avenue for AI‐assisted tools in the thoracic domain.

### 3.2 Generative AI in Specialized Surgical Tasks With Limited Sample Sizes

A cornerstone of LungSurg's methodological innovation is the pioneering use of a generative pre‐training (GPT) strategy to address the “small data” problem inherent in specialized surgical AI. The development of high‐performance models for procedures like VATS lobectomy is often constrained by the limited availability of expert‐annotated data due to the high cost and labor involved [19]. To overcome this bottleneck, we introduce a self‐supervised approach that leverages a vast corpus of unlabeled surgical videos. Specifically, we employ an MAE to pre‐train our model on a generative task–image inpainting–where the model learns to reconstruct randomly occluded regions of a surgical scene [20] (Figure S4).

This pre‐training regimen forces the model to develop a deep, contextual understanding of thoracic anatomy and the spatial relationships between instruments and tissues, effectively building a foundational “visual knowledge base” without any manual labels. It is crucial to distinguish this approach from generative data augmentation, where models are used to create synthetic images. Instead, our method uses a generative task to learn robust representations from real data. As confirmed by our ablation studies (Table S4), this strategy provides a significantly more powerful feature initialization than conventional ImageNet pre‐training or training from scratch. When subsequently fine‐tuned on our limited annotated dataset, the pre‐trained model achieves superior segmentation accuracy. This demonstrates a powerful and scalable paradigm for developing expert‐level AI in data‐scarce medical domains, marking a significant advancement over the reliance on purely supervised learning in prior surgical AI research [21].

### 3.3 Enhancing Surgical Education and Intraoperative Collaboration

The educational experiment within our study highlights LungSurg's significant potential to transform surgical training. Surgical education traditionally relies on a combination of didactic learning and hands‐on experience, both of which are limited by factors such as access to mentors and variability in surgical exposure [22]. LungSurg addresses these limitations by offering interactive visualizations and feedback, thereby enhancing the learning experience for surgical residents. The observed improvement in anatomical structure identification and surgical phase classification among residents who utilized LungSurg underscores its efficacy as an educational tool.

Moreover, LungSurg's ability to provide automatic identification of surgical phases and anatomical landmarks can foster better intraoperative collaboration. Effective teamwork and communication are paramount for optimal patient outcomes, particularly in complex surgeries where swift and accurate decision‐making is critical [23]. By keeping all team members informed of the current procedural step and anticipated actions, LungSurg facilitates a more coordinated surgical environment, aiming to enhance overall surgical efficiency.

### 3.4 Insights From Comparative Analysis With Human Experts

Our comparative analysis between LungSurg and human experts revealed that the AI system performs at a level comparable to senior surgeons in detecting vital anatomical structures. While senior surgeons exhibited higher precision (PIC), LungSurg demonstrated greater sensitivity (SID), suggesting its ability to identify a broader range of relevant features. This balance between precision and sensitivity is crucial in surgical settings, where the identification of all relevant anatomical structures is essential to avoid inadvertent injuries.

The high sensitivity of LungSurg may serve as a valuable adjunct to surgical practice, acting as an additional layer of safety by alerting surgeons to anatomical details that might otherwise be overlooked, especially in high‐stress or fast‐paced operative environments.

### 3.5 Innovative Algorithmic Approaches

The development of LungSurg incorporated several innovative algorithmic strategies that contribute to its high performance. The use of image inpainting techniques allowed the AI to reconstruct occluded or missing parts of the surgical field, thereby enhancing its ability to interpret complex scenes where instruments or tissue may obscure critical anatomy [24, 25]. This approach is particularly valuable in thoracic surgery, where factors such as smoke, blood, and rapid instrument movements frequently challenge visual clarity.

In addition, the integration of a co‐occurrence loss function based on the probability of object pairs improved the model's contextual understanding [26]. By considering the relationships between surgical instruments and anatomical structures, LungSurg achieved more accurate segmentation and classification within the surgical field. The combination of spatial and temporal networks in the SurgClass module enabled effective classification of surgical phases, capturing both the static and dynamic aspects of the procedure.

These algorithmic innovations not only enhance the current performance of LungSurg but also set a foundation for future AI‐driven advancements in surgical technology. The adaptability of these techniques suggests potential applications beyond VATS lobectomy, extending to other complex surgical procedures where anatomical variability and dynamic intraoperative conditions pose significant challenges.

### 3.6 Limitations and Future Directions

While LungSurg has demonstrated strong performance, its limitations primarily concern model generalizability and robustness, many of which are inherent to our strategic, phased research design. On a macroscopic level, our initial dataset deliberately excluded highly complex clinical cases involving severe adhesions, significant bleeding, or major anatomical anomalies. This was a methodical choice to first establish a robust baseline on standardized procedures. Consequently, the model's generalizability to these high‐difficulty scenarios remains a key focus for future work, where we will expand our dataset and develop specialized modules using techniques like anomaly detection. On a more granular level, even within standard procedures, the model's robustness can be challenged by adverse visual conditions.

As illustrated by typical failure cases (Figure S5), performance can degrade in scenarios with lens blur, heavy smoke, or rapid instrument movements. Future work will therefore also focus on enhancing model resilience through advanced image processing algorithms and the integration of temporal consistency checks. This dual approach–expanding clinical scope while reinforcing technical resilience–is critical for safely translating surgical AI into the high‐stakes environment of thoracic surgery.

Another major avenue for future research is the transition from an offline analysis tool to a real‐time intraoperative guidance system. This presents several interconnected challenges. First, the current system is not optimized for low latency. Achieving real‐time performance will require significant algorithm optimization through techniques like model pruning, quantization, or knowledge distillation to create a “lightweight” model. Second, effective real‐time integration necessitates seamless interoperability with existing surgical platforms and, critically, the design of intuitive user interfaces that enhance, rather than disrupt, the surgeon's workflow. Addressing these technical and human‐factors challenges through user‐centered design will be paramount for translating our system into a valuable tool for live surgery.

Similarly, our educational validation, while promising, was conducted as a pilot study. Although the results were statistically significant, the sample size was modest, and the participants were from a single institution. Furthermore, this study was limited by its focus on short‐term knowledge acquisition. Future larger‐scale, multi‐center trials are needed to confirm these educational benefits and, crucially, to assess long‐term skill retention and the transfer of these cognitive skills to practical performance in simulated or real operating room environments.

## 4 Conclusion

We have developed and validated LungSurg, an advanced AI system capable of expert‐level recognition of critical anatomical structures and surgical phases in VATS lobectomy. By integrating innovative techniques such as image inpainting and contextual learning, LungSurg addresses the challenges posed by anatomical variability and intraoperative occlusions inherent in thoracic surgery. Our multicenter study demonstrates the system's robustness, accuracy, and potential to transform surgical education and practice.

## 5 Methods

### 5.1 Study Design and Dataset Collection

This multicenter study was conducted across eight centers and approved by the Ethics Committee of the National Center for Respiratory Medicine (IRB number: 2022 NO.70). Given the retrospective nature of the study and the use of de‐identified video data, the requirement for individual patient informed consent was waived by the ethics committee. Our objective was to develop a dual‐function AI network capable of identifying (1) surgical structures (instruments and intrathoracic anatomy) and (2) surgical phases in VATS lobectomy procedures (Figure 1).

Between January 1, 2018, and January 1, 2024, we collected 222 integrated VATS lobectomy videos from eight centers encompassing complete surgical procedures–from endoscope insertion to wound closure. All surgeries were performed by senior thoracic surgeons, and the videos were reviewed by two experts (J.X.H. and W.W.) for quality assurance.

To build robust training and validation datasets, we allocated 165 videos from four centers for AI model development and reserved 57 videos from other centers for independent external validation (Figure 1A). These videos included procedures involving all five lung lobes, ensuring comprehensive coverage of surgical scenarios. To focus on standard surgical practices, we excluded videos featuring unconventional cases such as severe thoracic adhesions, significant intraoperative bleeding, complex lymph node dissections, or advanced procedures like sleeve lobectomy. The video data were collected from various endoscopic systems, including 2D and 3D laparoscopes from multiple manufacturers (e.g., Karl Storz, Stryker, Olympus), ensuring hardware heterogeneity in the dataset.

### 5.2 Video Annotation and Dataset Preparation

Patient identifiers were removed from all videos to maintain confidentiality. We extracted frames at a rate of one fps, sequentially numbering each frame. To manage the extensive annotation workload and enhance scene diversity, we adjusted the annotation frequency based on scene complexity.

Six certified thoracic surgeons, divided into two groups, performed the annotations. Videos were randomly allocated to each group in a 1:1 ratio. Within each group, annotations were cross‐validated to achieve consensus and minimize subjectivity. Using Labelme software (Massachusetts Institute of Technology, USA) [27], surgeons outlined and labeled surgical instruments and anatomical structures, creating a comprehensive segmentation dataset. Concurrently, we established a phase classification database by recording specific surgical phases along with corresponding start and end frame numbers.

We calculated Cohen's kappa scores between the two groups to assess intergroup annotation consistency and evaluate the impact of subjectivity on data quality. Detailed annotation protocols are provided in the Supporting Information‐Annotation framework for surgical video.

### 5.3 Identification of Surgical Structures and Phases

**FIGURE 6:**
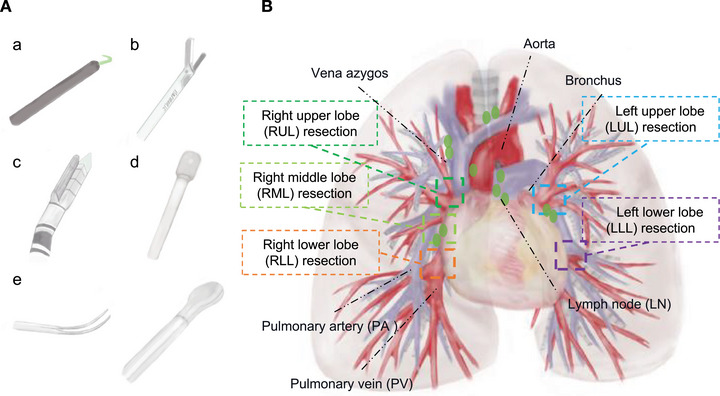
Demonstration of the annotated surgical structures and surgical phase involved in the Video‐assisted Thoracoscopic Lobectomy. (A) Presentation of the instruments. a Electrocoagulation; b Ultrasonic knife; c Stapler; d Suction; e Clamp. (B) Presentation of the thoracic anatomy and surgical areas relative to lobectomy with different phases. LLL, left lower lobe; LN, lymph node; LUL, right upper lobe; PA, pulmonary artery; PV, pulmonary vein; RLL, right lower lobe; RML, right middle lobe; RUL, right upper lobe.

We focused on commonly used surgical instruments and relevant anatomical structures in VATS lobectomy. The instruments annotated included staplers, suction devices, clamps, ultrasonic knives, and electrocautery tools (Figure 6A). The anatomical structures included the PA, PV, bronchus, LNs, and azygos vein on the right side, and the aorta on the left side (Figure 6B).

Given the absence of a standardized consensus on VATS lobectomy steps–which can exceed 20 distinct actions–we referred to the essential components outlined by the American Society of Thoracic Surgeons for training simulation and adapted them to our study into 14 actions (Table S7) [3, 28, 29, 30]. The surgical phases primarily included management of pulmonary bronchovascular structures, LNs, pulmonary fissures, and surgical field examination etc. The characteristics of the annotated data for the training set and the validation set are summarizes in Table S1.

### 5.4 AI Model Architecture and Training

We developed an AI system named *LungSurg*, featuring two main networks: *SurgSeg* for surgical structure identification and *SurgClass* for surgical phase classification. The annotated datasets for surgical structures and phases were used to train SurgSeg and SurgClass, respectively. SurgSeg first analyzes input video clips to generate segmentation masks, which are then utilized by SurgClass for phase classification–integrating both spatial and temporal information (Figure 1B).

### 5.5 SurgSeg Architecture and Training

To enhance scene diversity despite a finite number of annotations, we employed the image‐based instance segmentation model Mask R‐CNN [31] with a five‐stage ResNet‐50 backbone for SurgSeg. Recognizing that many target objects are slender and irregularly shaped, we incorporated a NL module [32] into the last three stages of the backbone to capture long‐range dependencies and enlarge the receptive field. In addition, inspired by the Bottleneck Transformer architecture, we modified the final stage using the MHSA module [33], further improving segmentation performance.

### 5.6 Innovative Pre‐Training With Image Inpainting

We introduced an innovative self‐supervised pre‐training strategy inspired by MAE [20] to learn robust feature representations from unlabeled data. In this paradigm, the model is trained on a generative task: reconstructing randomly occluded portions (up to 75%) of real, unlabeled surgical images (Figure S4). We term this “GPT” because the task's objective is inherently generative, forcing the model to develop a deep contextual understanding of surgical scenes–akin to how large language models are generatively pre‐trained–before being fine‐tuned on a specific downstream task. Crucially, this approach is distinct from using generative models (e.g., GANs) to augment datasets with entirely synthetic images. The powerful contextual representations learned during this phase were then fine‐tuned for the primary segmentation task.

To further leverage contextual dependencies between surgical instruments and anatomical structures, we calculated co‐occurrence probabilities between object pairs and introduced a novel co‐occurrence loss function during the fine‐tuning stage (Figure S6). This encouraged the model to consider relational information, enhancing segmentation accuracy.

The final SurgSeg model was trained by combining five loss functions: Region Proposal Network (RPN) loss (smooth L1 loss and binary cross‐entropy loss), Fast R‐CNN loss [34] (co‐occurrence loss and smooth L1 loss), and Mask R‐CNN loss [31] (binary cross‐entropy loss). We used the Adam optimizer with an initial learning rate of 0.0001. During the first 20 epochs, the weights of the pre‐trained encoder were frozen to stabilize training, after which all layers were unfrozen for end‐to‐end optimization. Images were resized to 512 × 512 pixels, and training was conducted with a batch size of eight on Tesla V100 GPUs.

### 5.7 SurgClass Architecture and Training

To enable SurgClass to effectively utilize knowledge from the segmentation task, we integrated four modalities of information from SurgSeg (Figure 1B):

1. Per‐pixel confidence scores from SurgSeg.
2. Dot product results between the original image and SurgSeg's segmentation mask.
3. Segmentation features from SurgSeg's encoder.
4. Pre‐trained image features from the encoder are used in pre‐training.

Features 1 and 2 were processed using a 3D action recognition network based on MobileNet_V3 [35], capturing spatial‐temporal patterns. Features 3 and 4 were input into a bidirectional long short‐term memory (bi‐LSTM) network [36] to model temporal dependencies. An MLP classifier then integrated outputs from both networks to produce the final phase classification for each video segment.

SurgClass was trained in two modes:

1. *Local mode*: Classifying 4–8‐s video segments to determine the corresponding surgical phase.
2. *Global mode*: Performing sliding window analysis across the entire surgery to output temporal segmentation of phases and transition probabilities.

Training utilized the Adam optimizer with a learning rate of 0.0001. If performance plateaued within three epochs, we switched to a multi‐class focal loss function to address class imbalance. Images were resized to 256 × 256 pixels, and training was conducted with a batch size of 16 on four Tesla V100 GPUs.

### 5.8 LungSurg‐Assisted Surgical Education

To assess LungSurg's educational potential, we conducted a prospective, single‐blind, pilot randomized controlled study. The written informed consent was obtained from all participants.

A total of 20 first‐year surgical residents with no prior specialized training in thoracic surgery were recruited to ensure a homogenous baseline. Participants were randomly assigned (1:1 ratio) via a computer‐generated sequence to either a control group or an experimental group. The assessors who graded the post‐training tests were blinded to the group allocations. Both groups followed a 1‐week structured curriculum (2 h/day). The control group (*n* = 10) used conventional learning resources (textbooks and standard surgical videos). The experimental group (*n* = 10) had access to the same resources, supplemented with the interactive LungSurg platform, which provides AI‐powered segmentation overlays and phase classification.

Post‐training performance was evaluated using a standardized, unseen test set. The assessment consisted of two tasks:

Anatomical and Instrument Identification: Participants were shown 50 static surgical screenshots and asked to identify pre‐specified anatomical structures and surgical instruments. An identification was scored as correct only if the object was both correctly labeled and accurately localized. The primary metric was identification accuracy, calculated separately for anatomical structures and instruments. Surgical Phase Classification: Participants viewed fifty 8‐s video clips and identified the corresponding surgical phase from a 14‐option multiple‐choice list. The primary metric was classification accuracy.

The accuracy rates between the two groups were compared using the chi‐squared (*χ* ^2^) test for independence to analyze the association between group allocation and the correctness of responses. For any analyses where the expected frequency in any cell of the contingency table was less than 5, Fisher's exact test was used instead. Given the pilot nature of this study, results were primarily interpreted based on effect sizes and 95% confidence intervals, with *p*‐values provided for reference.

### 5.9 Statistical Analysis

For evaluating SurgSeg's performance, we employed the IoU metric: (Label_Mask ∩ Prediction_Mask) / (Label_Mask U Prediction_Mask). A threshold of IoU > 0.5 was considered correct segmentation. The mAP at IoU > 0.5 was calculated as the average area under the Precision‐Recall curve for each category. SurgClass's performance was assessed using Top‐1 and Top‐3 accuracy metrics for phase classification on each video frame.

All statistical analyses were performed using Python 3.8. Visualizations were created using Matplotlib. Labelme software (Massachusetts Institute of Technology, USA) was used to finish the labeling of the surgical elements.

## Author Contributions

H.L. gave conception and design of this study. L.Z., Y.G., and J.H. offered the administrative support. H.L., Z.Y., and Yudong Z. developed the *LungSurg* system. K.D., H.L., J.S., P.L., J.J., G.Z., X.Z., and H.C. assisted to improve the system. H.L., and Z.Y. conducted experimental analysis. H.Z., Yuzhuo Z., S.L., M.C., X.W., A.R., and W.W. leaded the data collection. H.L., Z.Y., L.Z., Y.G., and J.H. wrote the manuscript. H.L. has accessed and verified the data. H.L., L.Z., Y.G., and J.H. were responsible for the decision to submit the manuscript. All authors read and approved the manuscript.

## Funding

This work was supported by the Grant R&D Program of Guangzhou National Laboratory (No. SRPG22‐017), 2025 Engineering Science and Technology Academic Symposium Project of the Chinese Academy of Engineering (2025‐XS‐22), and the National Natural Science Foundation of China (No. 82503973). The study sponsors had no role in the study design; in the collection, analysis, and interpretation of data; in the writing of the report; or in the decision to submit the paper for publication.

## Ethics Statement

This multicenter study was conducted across eight centers and approved by the Ethics Committee of the National Center for Respiratory Medicine (IRB number: 2022 NO.70). Given the retrospective nature of the study and the use of de‐identified video data, the requirement for individual patient informed consent was waived by the ethics committee.

## Conflicts of Interest

The authors declare no conflicts of interest.

## Supporting information




**Table S1a**: Annotation data characteristics for training set
**Table S1b**: Annotation data characteristics for validation set
**Table S2** The overall segmentation performance of the LungSurg trained in a single center and validated on the external validation set
**Table S3**: Performance of LungSurg on the External Validation Set, Stratified by Center
**Table S4**: Ablation experiments with the ***SurgSeg*** in internal validation
**Table S5**: The overall classification performance of the ***LungSurg*** trained in a single center on the external validation set.
**Table S6**: Ablation experiment results of ***SurgClass*** (TOP1 and TOP3 ACC for each frame in the internal validation set)
**Table S7**: Definition of each phase of Video‐assisted thoracoscopic (VATS) lobectomy
**Figure S1**: Duration of each surgical phase in the left (A) and right (B) lung
**Figure S2**: Visual assessment of the model's segmentation performance on surgical scenes from external validation centers
**Figure S3**: Ablation study on segmentation task
**Figure S4**: Image filling task in pre‐training
**Figure S5**: Examples of incorrect segmentation and classification
**Figure S6**: Co‐occurrence loss of each annotation in the left and the right VATS lobectomy
**Supplementary** ‐Annotation framework for surgical video

**Supporting File 1**: mco270613‐sup‐0002‐DemoS1.mp4.

**Supporting File 2**: mco270613‐sup‐0002‐DemoS2.mp4.

## Data Availability

To promote reproducibility and further research, we have made most of the core source code of the LungSurg model open‐source (facilitating the reproduction of the entire network architecture and training process), and a portion of the annotated validation dataset is also provided. These open‐source materials can be downloaded via a Baidu Netdisk shared link (SurgeryAI: https://pan.baidu.com/s/1Hfy3QO2oNWL4nLlV5y_m2A; Passcode: 1234). The complete training process records and full raw video data contain sensitive patient information and thus cannot be fully made public. However, they are available from the corresponding author (drjianxing.he@gmail.com) upon reasonable request, contingent upon the signing of a Data Use Agreement to ensure patient privacy.

## References

[1] H. Sung , J. Ferlay , R. L. Siegel , et al., “Global Cancer Statistics 2020: GLOBOCAN Estimates of Incidence and Mortality Worldwide for 36 Cancers in 185 Countries,” CA: A Cancer Journal for Clinicians 71, no. 3 (2021): 209–249. 33538338 10.3322/caac.21660

[2] T. A. D'Amico , “The Video‐Assisted Thoracoscopic or Open Lobectomy (VIOLET) Trial: The Final Chapter to This Epic,” Journal of Thoracic and Cardiovascular Surgery 166, no. 1 (2023): 265–267. 36774208 10.1016/j.jtcvs.2022.12.022

[3] T. A. Haidari , L. J. Nayahangan , F. Bjerrum , et al., “Consensus on Technical Procedures for Simulation‐Based Training in Thoracic Surgery: An International Needs Assessment,” European Journal of Cardio‐Thoracic Surgery 63, no. 4 (2023): ezad058. 36808223 10.1093/ejcts/ezad058

[4] A. Vieira , E. Bourdages‐Pageau , K. Kennedy , and P. A. Ugalde , “The Learning Curve on Uniportal Video‐Assisted Thoracic Surgery: An Analysis of Proficiency,” Journal of Thoracic and Cardiovascular Surgery 159, no. 6 (2020): 2487–2495.e2. 31926696 10.1016/j.jtcvs.2019.11.006

[5] S. Grossi , M. Cattoni , N. Rotolo , and A. Imperatori , “Video‐Assisted Thoracoscopic Surgery Simulation and Training: A Comprehensive Literature Review,” BMC Medical Education 23, no. 1 (2023): 535. 37501111 10.1186/s12909-023-04482-z PMC10375656

[6] Y. Li , N. Raison , S. Ourselin , T. Mahmoodi , P. Dasgupta , and A. Granados , “AI Solutions for Overcoming Delays in Telesurgery and Telementoring to Enhance Surgical Practice and Education,” Journal of Robotic Surgery 18, no. 1 (2024): 403. 39527379 10.1007/s11701-024-02153-9 PMC11554828

[7] D. A. Hashimoto , G. Rosman , D. Rus , and O. R. Meireles , “Artificial Intelligence in Surgery: Promises and Perils,” Annals of Surgery 268, no. 1 (2018): 70–76. 29389679 10.1097/SLA.0000000000002693 PMC5995666

[8] T. M. Ward , D. A. Hashimoto , Y. Ban , et al., “Automated Operative Phase Identification in Peroral Endoscopic Myotomy,” Surgical Endoscopy 35, no. 7 (2021): 4008–4015. 32720177 10.1007/s00464-020-07833-9 PMC7854950

[9] R. Garcia Nespolo , D. Yi , E. Cole , N. Valikodath , C. Luciano , and Y. I. Leiderman , “Evaluation of Artificial Intelligence‐Based Intraoperative Guidance Tools for Phacoemulsification Cataract Surgery,” JAMA Ophthalmology 140, no. 2 (2022): 170–177. 35024773 10.1001/jamaophthalmol.2021.5742 PMC8855235

[10] P. Mascagni , A. Vardazaryan , D. Alapatt , et al., “Artificial Intelligence for Surgical Safety: Automatic Assessment of the Critical View of Safety in Laparoscopic Cholecystectomy Using Deep Learning,” Annals of Surgery 275, no. 5 (2022): 955–961. 33201104 10.1097/SLA.0000000000004351

[11] S. Wu , Z. Chen , R. Liu , et al., “SurgSmart: An Artificial Intelligent System for Quality Control in Laparoscopic Cholecystectomy: An Observational Study,” International Journal of Surgery 109, no. 5 (2023): 1105–1114. 37039533 10.1097/JS9.0000000000000329 PMC10389595

[12] Z. Chen , J. An , S. Wu , et al., “Surgesture: A Novel Instrument Based on Surgical Actions for Objective Skill Assessment,” Surgical Endoscopy 36, no. 8 (2022): 6113–6121. 35737138 10.1007/s00464-022-09108-x

[13] Z. Chen , D. Yang , A. Li , et al., “Decoding Surgical Skill: An Objective and Efficient Algorithm for Surgical Skill Classification Based on Surgical Gesture Features‐Experimental Studies,” International Journal of Surgery 110, no. 3 (2024): 1441–1449. 38079605 10.1097/JS9.0000000000000975 PMC10942222

[14] Z. Chen , Y. Zhang , Z. Yan , et al., “Artificial Intelligence Assisted Display in Thoracic Surgery: Development and Possibilities,” Journal of Thoracic Disease 13, no. 12 (2021): 6994–7005. 35070382 10.21037/jtd-21-1240 PMC8743398

[15] O. Elharrouss , N. Almaadeed , S. Al‐Maadeed , and Y. Akbari , “Image Inpainting: A Review,” Neural Processing Letters 51 (2020): 2007–2028.

[16] S. Wu , M. Tang , J. Liu , et al., “Impact of an AI‐Based Laparoscopic Cholecystectomy Coaching Program on the Surgical Performance: A Randomized Controlled Trial,” International Journal of Surgery 110, no. 12 (2024): 7816–7823. 38896869 10.1097/JS9.0000000000001798 PMC11634122

[17] P. Mascagni , D. Alapatt , A. Lapergola , et al., “Early‐Stage Clinical Evaluation of Real‐Time Artificial Intelligence Assistance for Laparoscopic Cholecystectomy,” British Journal of Surgery 111, no. 1 (2024): znad353. 37935636 10.1093/bjs/znad353

[18] M. Kawamura , Y. Endo , A. Fujinaga , et al., “Development of an Artificial Intelligence System for Real‐Time Intraoperative Assessment of the Critical View of Safety in Laparoscopic Cholecystectomy,” Surgical Endoscopy 37, no. 11 (2023): 8755–8763. 37567981 10.1007/s00464-023-10328-y

[19] S. Sai , A. Gaur , R. Sai , V. Chamola , M. Guizani , and J. J. Rodrigues , “Generative AI for Transformative Healthcare: A Comprehensive Study of Emerging Models, Applications, Case Studies and Limitations,” IEEE Access 2 (2024): 31078–31106.

[20] K. He , X. Chen , S. Xie , Y. Li , P. Dollár , and R. Girshick , “Masked Autoencoders Are Scalable Vision Learners,” in Proceedings of the IEEE/CVF Conference on Computer Vision and Pattern Recognition , (CVPR, 2022), 16000–16009.

[21] Y. Chen , X.‐H. Yang , Z. Wei , et al., “Generative Adversarial Networks in Medical Image Augmentation: A Review,” Computers in Biology and Medicine 144 (2022): 105382. 35276550 10.1016/j.compbiomed.2022.105382

[22] M. S. Hameed , S. Laplante , C. Masino , et al., “What Is the Educational Value and Clinical Utility of Artificial Intelligence for Intraoperative and Postoperative Video Analysis? A Survey of Surgeons and Trainees,” Surgical Endoscopy 37, no. 12 (2023): 9453–9460. 37697116 10.1007/s00464-023-10377-3

[23] M. U. Khalid , S. Laplante , C. Masino , et al., “Use of Artificial Intelligence for Decision‐Support to Avoid High‐Risk Behaviors During Laparoscopic Cholecystectomy,” Surgical Endoscopy 37, no. 12 (2023): 9467–9475. 37697115 10.1007/s00464-023-10403-4

[24] J. Jam , C. Kendrick , K. Walker , V. Drouard , J. G. Hsu , and M. H. Yap , “A Comprehensive Review of Past and Present Image Inpainting Methods,” Computer Vision and Image Understanding 203 (2021): 103147.

[25] X. Zhang , D. Zhai , T. Li , Y. Zhou , and Y. Lin , “Image Inpainting Based on Deep Learning: A Review,” Information Fusion 90 (2023): 74–94.

[26] J. Jiao , C. Yin , and F. Teng , “W‐Net: Deep Convolutional Network With Gray‐Level Co‐Occurrence Matrix and Hybrid Loss Function for Hyperspectral Image Classification,” in Advanced Intelligent Computing Technology and Applications, (Springer, 2023), 112–124.

[27] B. C. Russell , A. Torralba , K. P. Murphy , and W. T. Freeman , “LabelMe: A Database and Web‐Based Tool for Image Annotation,” International Journal of Computer Vision 77 (2008): 157–173.

[28] M. K. Ferguson and C. Bennett , “Identification of Essential Components of Thoracoscopic Lobectomy and Targets for Simulation,” Annals of Thoracic Surgery 103, no. 4 (2017): 1322–1329. 28267978 10.1016/j.athoracsur.2016.12.021

[29] D. S. Bryan , M. K. Ferguson , M. B. Antonoff , et al., “Consensus for Thoracoscopic Left Upper Lobectomy‐Essential Components and Targets for Simulation,” Annals of Thoracic Surgery 112, no. 2 (2021): 436–442. 33127408 10.1016/j.athoracsur.2020.06.152

[30] P. A. Erwin , A. C. Lee , U. Ahmad , et al., “Consensus for Thoracoscopic Lower Lobectomy: Essential Components and Targets for Simulation,” Annals of Thoracic Surgery 114, no. 5 (2022): 1895–1901. 34688617 10.1016/j.athoracsur.2021.09.033

[31] K. He , G. Gkioxari , P. Dollár , and R. Girshick , “Mask R‐CNN,” in Proceedings of the IEEE/CVF International Conference on Computer Vision and Pattern Recognition , (CVPR, 2017), 2961–2969.

[32] X. Wang , R. Girshick , A. Gupta , and K. He , “Non‐Local Neural Networks,” in Proceedings of the IEEE/CVF International Conference on Computer Vision and Pattern Recognition , (CVPR, 2018), 7794–7803.

[33] A. Srinivas , T.‐Y. Lin , N. Parmar , J. Shlens , P. Abbeel , and A. Vaswani , “Bottleneck Transformers for Visual Recognition,” in Proceedings of the IEEE/CVF International Conference on Computer Vision and Pattern Recognition , (CVPR, 2021), 16519–16529.

[34] S. Ren , K. He , R. Girshick , and J. Sun , “Faster R‐CNN: Towards Real‐Time Object Detection With Region Proposal Networks,” preprint, arXiv, June 4, 2015. 10.1109/TPAMI.2016.2577031 27295650

[35] A. Howard , M. Sandler , G. Chu , et al., “Searching for Mobilenetv3,” in Proceedings of the IEEE International Conference on Computer Vision , (ICCV, 2019), 1314–1324.

[36] P. Zhou , W. Shi , J. Tian , et al., “Attention‐Based Bidirectional Long Short‐Term Memory Networks for Relation Classification,” in Proceedings of the 54th Annual Meeting of the Association for Computational Linguistics , ed. K. Erk and N. A. Smith , (Association for Computational Linguistics, 2016), 207–212.

