# Supplementary material for: LungSurg: A Generative AI System for Segmentation and Phase Classification in Thoracoscopic Lobectomy
Source: MedComm (2020). 2026 Jan 20;7(2):e70613. doi: 10.1002/mco2.70613 (PMC12820416; doi:10.1002/mco2.70613)

**Title**

**LungSurg: A Generative AI System for Segmentation and Phase Classification in Thoracoscopic Lobectomy**

**Supplementary Materials**

**1-Table S1a** Annotation data characteristics for training set

**2-Table S1b** Annotation data characteristics for validation set

**3-Table S2** The overall segmentation performance of the LungSurg trained in a single center and validated on the external validation set

**4-Table S3** Performance of LungSurg on the External Validation Set, Stratified by Center

**5-Table S4** Ablation experiments with the ***SurgSeg*** in internal validation

**6-Table S5** The overall classification performance of the ***LungSurg*** trained in a single center on the external validation set.

**7-Table S6** Ablation experiment results of ***SurgClass*** (TOP1 and TOP3 ACC for each frame in the internal validation set)

**8-Table S7** Definition of each phase of Video-assisted thoracoscopic (VATS) lobectomy

**9-Figure S1** Duration of each surgical phase in the left (A) and right (B) lung

**10-Figure S2** Visual assessment of the model's segmentation performance on surgical scenes from external validation centers

**11-Figure S3** Ablation study on segmentation task

**12-Figure S4** Image filling task in pre-training

**13-Figure S5** Examples of incorrect segmentation and classification

**14-Figure S6** Co-occurrence loss of each annotation in the left and the right VATS lobectomy

**15-Supplementary**-Annotation framework for surgical video

**Table S1a** Annotation data characteristics for training set

| Number | Overall | Internal training set | | | | |  | External training set | | | | |
| --- | --- | --- | --- | --- | --- | --- | --- | --- | --- | --- | --- | --- |
|  |  | LUL | LLL | RUL | RLL | RML |  | LUL | LLL | RUL | RLL | RML |
| **Video count (segmentation/ classification task)** | **165** | 34 | 23 | 40 | 23 | 3 |  | 7 | 2 | 22 | 5 | 6 |
| **Annotated frames count (n)** | **25260** | 7824 | 3918 | 6276 | 2908 | 512 |  | 710 | 200 | 2010 | 351 | 551 |
|  |  |  |  |  |  |  |  |  |  |  |  |  |
| ***Surgical structure categories (n)*** |  |  |  |  |  |  |  |  |  |  |  |  |
| Electrocoagulation | 25517 | 9343 | 3239 | 6078 | 2806 | 365 |  | 658 | 196 | 2000 | 348 | 484 |
| Ultrasonic knife | 17917 | 4489 | 3253 | 4884 | 1974 | 327 |  | 323 | 108 | 1808 | 304 | 447 |
| Stapler | 14996 | 5138 | 1984 | 3247 | 1197 | 305 |  | 584 | 117 | 1771 | 243 | 410 |
| Suction | 75502 | 24732 | 11375 | 17632 | 9450 | 1676 |  | 2089 | 712 | 5866 | 789 | 1181 |
| Clamp | 67016 | 22300 | 11666 | 14169 | 7255 | 1129 |  | 1998 | 896 | 4977 | 1086 | 1540 |
| Pulmonary artery (PA) | 86067 | 29492 | 12500 | 21370 | 7915 | 1428 |  | 2873 | 1000 | 6893 | 1085 | 1511 |
| Pulmonary vein (PV) | 54523 | 20379 | 6041 | 13595 | 4224 | 1444 |  | 1997 | 512 | 4421 | 554 | 1356 |
| Bronchus (B) | 46888 | 13676 | 7259 | 12338 | 5282 | 891 |  | 1279 | 507 | 4616 | 412 | 628 |
| Lymph node (LN) | 58915 | 17101 | 10550 | 12227 | 6790 | 2424 |  | 1854 | 903 | 3995 | 741 | 2330 |
| Vena azygos | 19810 | 0 | 0 | 13965 | 1664 | 183 |  | 0 | 0 | 3490 | 217 | 291 |
| aorta | 34875 | 20858 | 10768 | 0 | 0 | 0 |  | 2216 | 1033 | 0 | 0 | 0 |
|  |  |  |  |  |  |  |  |  |  |  |  |  |
| ***Surgical phases (n / seconds)*** |  |  |  |  |  |  |  |  |  |  |  |  |
| Wedge resection | 25690 | 4202 | 4440 | 5410 | 3413 | 1017 |  | 996 | 420 | 2311 | 1152 | 2329 |
| Improve the mobility of the lung by dissecting ligament/adhesion band | 34980 | 6413 | 7955 | 7241 | 5677 | 513 |  | 2102 | 673 | 2014 | 1415 | 977 |
| Management of the pulmonary vein | 66890 | 16718 | 8073 | 16238 | 6009 | 1049 |  | 4233 | 661 | 10472 | 1330 | 2107 |
| Management of the fissure | 53847 | 12870 | 9271 | 6385 | 12216 | 944 |  | 2224 | 862 | 4086 | 3330 | 1659 |
| Management of the pulmonary artery | 98190 | 36637 | 10146 | 21063 | 13307 | 1509 |  | 7433 | 999 | 1925 | 2973 | 2198 |
| Management of the lymph nodes | 166984 | 22264 | 15542 | 50264 | 30401 | 1904 |  | 4008 | 1555 | 30447 | 6199 | 4410 |
| Operative field examination / bleeding prevention management | 202350 | 47376 | 25557 | 46857 | 26957 | 3963 |  | 12099 | 3341 | 24155 | 4278 | 7767 |
| Management of the bronchus | 57143 | 16117 | 7729 | 10467 | 9073 | 594 |  | 3001 | 774 | 5911 | 2568 | 909 |
| Resection the targeted lung lobe | 47059 | 9540 | 6084 | 15064 | 4841 | 251 |  | 2075 | 708 | 6797 | 1199 | 500 |
| Leakage examination/Operative field cleanness | 33408 | 7105 | 4306 | 6821 | 8350 | 604 |  | 1096 | 477 | 2254 | 1253 | 1142 |
| Nerve block | 12765 | 1964 | 1985 | 2647 | 1558 | 90 |  | 355 | 254 | 1095 | 2746 | 71 |
| Extrathoracic scenes | 39258 | 8388 | 6064 | 9387 | 6540 | 108 |  | 1278 | 782 | 5261 | 1144 | 315 |
| Waiting | 10668 | 954 | 905 | 1643 | 714 | 232 |  | 2499 | 0 | 2951 | 664 | 106 |
| Lung Inflation for targeted bronchus identification | 3822 | 2098 | 0 | 319 | 255 | 0 |  | 98 | 0 | 0 | 1052 | 0 |
|  |  |  |  |  |  |  |  |  |  |  |  |  |

right upper lobe [RUL], right middle lobe [RML], right lower lobe [RLL], left upper lobe [LUL], and left lower lobe [LLL]

**Table S1b** Annotation data characteristics for validation set

| Number | Overall | Internal validation set | | | | | |  | | External validation set | | | | | |
| --- | --- | --- | --- | --- | --- | --- | --- | --- | --- | --- | --- | --- | --- | --- | --- |
|  |  | LUL | LLL | RUL | RLL | RML |  | | LUL | | LLL | RUL | RLL | RML |  |
| **Video count (segmentation/ classification task)** | **57** | 8 | 5 | 9 | 5 | 2 |  | | 6 | | 2 | 7 | 9 | 4 |  |
| **Annotated frames count (n)** | **6767** | 1956 | 980 | 1570 | 727 | 128 |  | | 302 | | 101 | 350 | 451 | 202 |  |
|  |  |  |  |  |  |  |  | |  | |  |  |  |  |  |
| ***Surgical structure categories (n)*** |  |  |  |  |  |  |  | |  | |  |  |  |  |  |
| Electrocoagulation | 7004 | 2300 | 820 | 1511 | 720 | 130 |  | | 339 | | 98 | 351 | 440 | 295 |  |
| Ultrasonic knife | 4577 | 1135 | 801 | 1233 | 454 | 77 |  | | 173 | | 87 | 188 | 275 | 154 |  |
| Stapler | 3983 | 1298 | 496 | 801 | 312 | 75 |  | | 256 | | 54 | 240 | 299 | 152 |  |
| Suction | 20361 | 6188 | 2834 | 4400 | 2366 | 440 |  | | 806 | | 345 | 951 | 1431 | 600 |  |
| Clamp | 17852 | 5575 | 2930 | 3555 | 1981 | 290 |  | | 748 | | 312 | 855 | 1058 | 548 |  |
| Pulmonary artery (PA) | 22169 | 7371 | 3125 | 5344 | 1977 | 356 |  | | 1115 | | 311 | 1158 | 984 | 428 |  |
| Pulmonary vein (PV) | 14425 | 5092 | 1511 | 3369 | 1057 | 344 |  | | 751 | | 186 | 719 | 883 | 513 |  |
| Bronchus (B) | 12663 | 3412 | 1827 | 3085 | 1320 | 221 |  | | 591 | | 234 | 606 | 1001 | 366 |  |
| Lymph node (LN) | 16359 | 4275 | 2634 | 3054 | 1698 | 608 |  | | 696 | | 379 | 611 | 1220 | 1184 |  |
| Vena azygos | 5011 | 0 | 0 | 3496 | 417 | 44 |  | | 0 | | 0 | 665 | 302 | 87 |  |
| aorta | 9118 | 5211 | 2692 | 0 | 0 | 0 |  | | 876 | | 339 | 0 | 0 | 0 |  |
|  |  |  |  |  |  |  |  | |  | |  |  |  |  |  |
| ***Surgical phases (n / seconds)*** |  |  |  |  |  |  |  | |  | |  |  |  |  |  |
| Wedge resection | 6664 | 285 | 1056 | 1381 | 931 | 0 |  | | 472 | | 105 | 871 | 1412 | 151 |  |
| Improve the mobility of the lung by dissecting ligament/adhesion band | 6566 | 956 | 752 | 1699 | 422 | 148 |  | | 866 | | 185 | 1143 | 251 | 144 |  |
| Management of the pulmonary vein | 28405 | 4859 | 2231 | 3588 | 1393 | 866 |  | | 4843 | | 1944 | 5214 | 2235 | 1232 |  |
| Management of the fissure | 21685 | 4121 | 2003 | 918 | 1956 | 365 |  | | 3886 | | 1151 | 2437 | 4421 | 427 |  |
| Management of the pulmonary artery | 26955 | 8506 | 2909 | 4838 | 4462 | 678 |  | | 1183 | | 591 | 872 | 2141 | 775 |  |
| Management of the lymph nodes | 48823 | 5368 | 3205 | 10702 | 8334 | 4121 |  | | 2493 | | 1286 | 3784 | 7778 | 1752 |  |
| Operative field examination / bleeding prevention management | 44410 | 11379 | 5057 | 7041 | 6452 | 1804 |  | | 1585 | | 568 | 3778 | 6125 | 621 |  |
| Management of the bronchus | 15639 | 2042 | 1425 | 2430 | 1723 | 364 |  | | 1397 | | 831 | 2434 | 2241 | 752 |  |
| Resection the targeted lung lobe | 10802 | 1640 | 1527 | 1660 | 1569 | 188 |  | | 904 | | 513 | 1170 | 1525 | 106 |  |
| Leakage examination/Operative field cleanness | 12528 | 2911 | 934 | 3466 | 1288 | 489 |  | | 685 | | 351 | 777 | 993 | 634 |  |
| Nerve block | 2634 | 489 | 278 | 823 | 172 | 0 |  | | 121 | | 0 | 588 | 163 | 0 |  |
| Extrathoracic scenes | 17944 | 1299 | 429 | 5029 | 1492 | 759 |  | | 1161 | | 520 | 3425 | 3321 | 509 |  |
| Waiting | 977 | 161 | 160 | 0 | 0 | 0 |  | | 142 | | 211 | 121 | 74 | 108 |  |
| Lung Inflation for targeted bronchus identification | 189 | 0 | 0 | 0 | 52 | 0 |  | | 0 | | 62 | 0 | 75 | 0 |  |
|  |  |  |  |  |  |  |  | |  | |  |  |  |  |  |

right upper lobe [RUL], right middle lobe [RML], right lower lobe [RLL], left upper lobe [LUL], and left lower lobe [LLL]

**Table S2** The overall segmentation performance of the ***LungSurg*** trained in a single center on the external validation set.

| Classes | Left | Right |
| --- | --- | --- |
| **Overall** | 0.602 | 0.558 |
| **Overall Instruments** | 0.680 | 0.567 |
| Electrocoagulation | 0.729 | 0.612 |
| Ultrasonic knife | 0.725 | 0.522 |
| Stapler | 0.813 | 0.617 |
| Suction | 0.575 | 0.633 |
| Clamp | 0.513 | 0.424 |
|  |  |  |
| **Overall Anatomy** | 0.529 | 0.551 |
| *Common features* |  |  |
| Pulmonary artery (PA) | 0.602 | 0.465 |
| Pulmonary vein (PV) | 0.500 | 0.634 |
| Bronchus (B) | 0.608 | 0.500 |
| Lymph node (LN) | 0.760 | 0.505 |
|  |  |  |
| *Right side* |  |  |
| Vena azygos | - | 0.682 |
|  |  |  |
| *Left side* |  |  |
| aorta | 0.632 | - |
| * The indicator was the mean average precision | | |

**Table S3** Performance of LungSurg on the External Validation Set, Stratified by Center

| Classes | external set 1 | external set 2 | external set 3 |
| --- | --- | --- | --- |
| **Overall** |  |  |  |
| Left | 0.751 | 0.753 | 0.726 |
| Right | 0.747 | 0.692 | 0.720 |
| **Overall Instruments** |  |  |  |
| Left | 0.829 | 0.837 | 0.744 |
| Right | 0.794 | 0.716 | 0.735 |
| **Overall Anatomy** |  |  |  |
| Left | 0.673 | 0.669 | 0.678 |
| Right | 0.689 | 0.670 | 0.675 |
| * The indicator was the mean average precision | | |  |

**Table S4** Ablation experiments with the ***SurgSeg*** in internal validation

| **Model** | **Left** | **Right** |
| --- | --- | --- |
| ***Baseline*** |  |  |
| ResNet50 | 0.766 | 0.672 |
|  |  |  |
| ***SurgSeg_Base*** |  |  |
| FPN | 0.768 | 0.685 |
| FPN + NL | 0.776 | 0.724 |
| FPN + NL + MHSA | 0.789 | 0.745 |
|  |  |  |
| ***SurgSeg_Turbo*** |  |  |
| Pretrain_rate 0.5 | 0.811 | 0.764 |
| Loss_co-occurrence | 0.809 | 0.769 |
| PreT+ Loss | 0.816 | 0.780 |

Feature Pyramid Network [FPN], non-local [NL], Multi-Head Self-Attention [MHSA]; Base (after upgrading the encoder), Turbo (incorporates pre-training and coexistence loss).

**Table S5** The overall classification performance of the ***LungSurg*** trained in a single center on the external validation set.

|  | **TOP 1** | **TOP 3** |
| --- | --- | --- |
| **Overall**  Wedge resection | 0.662  0.535 | 0.803  0.875 |
| Mobilize the lung by dissecting ligament/adhesion band | 0.694 | 0.752 |
| Management of the pulmonary vein | 0.681 | 0.708 |
| Management of the fissure | 0.710 | 0.850 |
| Management of the pulmonary artery | 0.699 | 0.744 |
| Management of the lymph nodes | 0.520 | 0.797 |
| Operative field management | 0.835 | 0.900 |
| Management of the bronchus | 0.678 | 0.778 |
| Resection the targeted lung lobe | 0.680 | 0.787 |
| Leakage examination/Operative field cleanness | 0.760 | 0.885 |
| Nerve block | 0.541 | 0.858 |
| Extrathoracic scenes | 0.775 | 0.875 |
| Waiting | 0.615 | 0.717 |
| Lung Inflation for targeted bronchus identification | 0.543 | 0.714 |

* The indicator was the accuracy

**Table S6** Ablation experiment results of ***SurgClass*** (Top1 and Top3 ACC for each frame in the internal validation set)

| **Model** | **Local Top1** | **Local Top3** | **Global Top1** | **Global Top3** |
| --- | --- | --- | --- | --- |
| **Action Net** |  |  |  |  |
| RGB Image | 0.582 | 0.723 | 0.612 | 0.757 |
| Image+SegMask | 0.650 | 0.809 | 0.673 | 0.870 |
|  |  |  |  |  |
| **Temporal Net** |  |  |  |  |
| Img Feature | 0.556 | 0.708 | 0.611 | 0.770 |
| Img&Seg Feature | 0.594 | 0.710 | 0.630 | 0.833 |
|  |  |  |  |  |
| **Final SurgClass** |  |  |  |  |
| A&T Net | 0.671 | 0.890 | 0.757 | 0.929 |

* The indicator was the ACC (accuracy); Local, local mode analysis (8-second clips); Global, global mode analysis (entire videos).

**Table S7** Definition of each phase of Video-assisted thoracoscopic (VATS) lobectomy

| **Surgical phases** | Start point | End point |
| --- | --- | --- |
| Nerve block | As the time the needle emerges and began to hit the intercostal nerve or the vagus nerve behind the superior vena cava | The needle withdrawal |
| Wedge resection | Presence of stapler | The excised lung tissue is taken out of the chest |
| Mobilize the lung by dissecting ligament/adhesion band | The cutting device appears and heads toward the connective tissue | Withdrawal of the instrument |
| Management of the fissure | Relevant instruments are present and ready to dissect, isolate, and divide the pulmonary fissure | Withdrawal of the instrument |
| Management of the pulmonary artery (PA) | Relevant instruments are present and ready to dissect, isolate, and divide the pulmonary artery | Withdrawal of the instrument |
| Management of the pulmonary vein (PV) | Relevant instruments are present and ready to dissect, isolate, and divide the pulmonary vein | Withdrawal of the instrument |
| Management of the bronchus | Relevant instruments are present and ready to dissect, isolate, and divide the bronchus | Withdrawal of the instrument |
| Management of the lymph nodes (LN) | Relevant instruments are present and ready to dissect the lymph nodes | Withdrawal of the instrument |
| Lung inflation for targeted bronchus identification | After the stapler is closed | Before the stapler is excitation |
| Resection the targeted lung lobe | Presence of stapler | The excised lung lobe is taken out of the chest |
| Leakage examination/operative field cleanness | Flush the chest cavity and ready to inflate the lungs | The chest cavity is basically drained |
| Operative field management | Instrument ready for hemostasis, ligation, clamp, placement of biological excipients | End of movements |
| Waiting | No related actions longer than 10-30 frames | Show action with definite meaning |
| Extrathoracic scenes | Thoracoscope draw out of thorax | Thoracoscopic insert into the chest |

**Figure S1A** Duration of each surgical phase in the left lung

**Figure S1B** Duration of each surgical phase in the left lung

**Figure S2** Visual assessment of the model's segmentation performance on surgical scenes from external validation centers

**
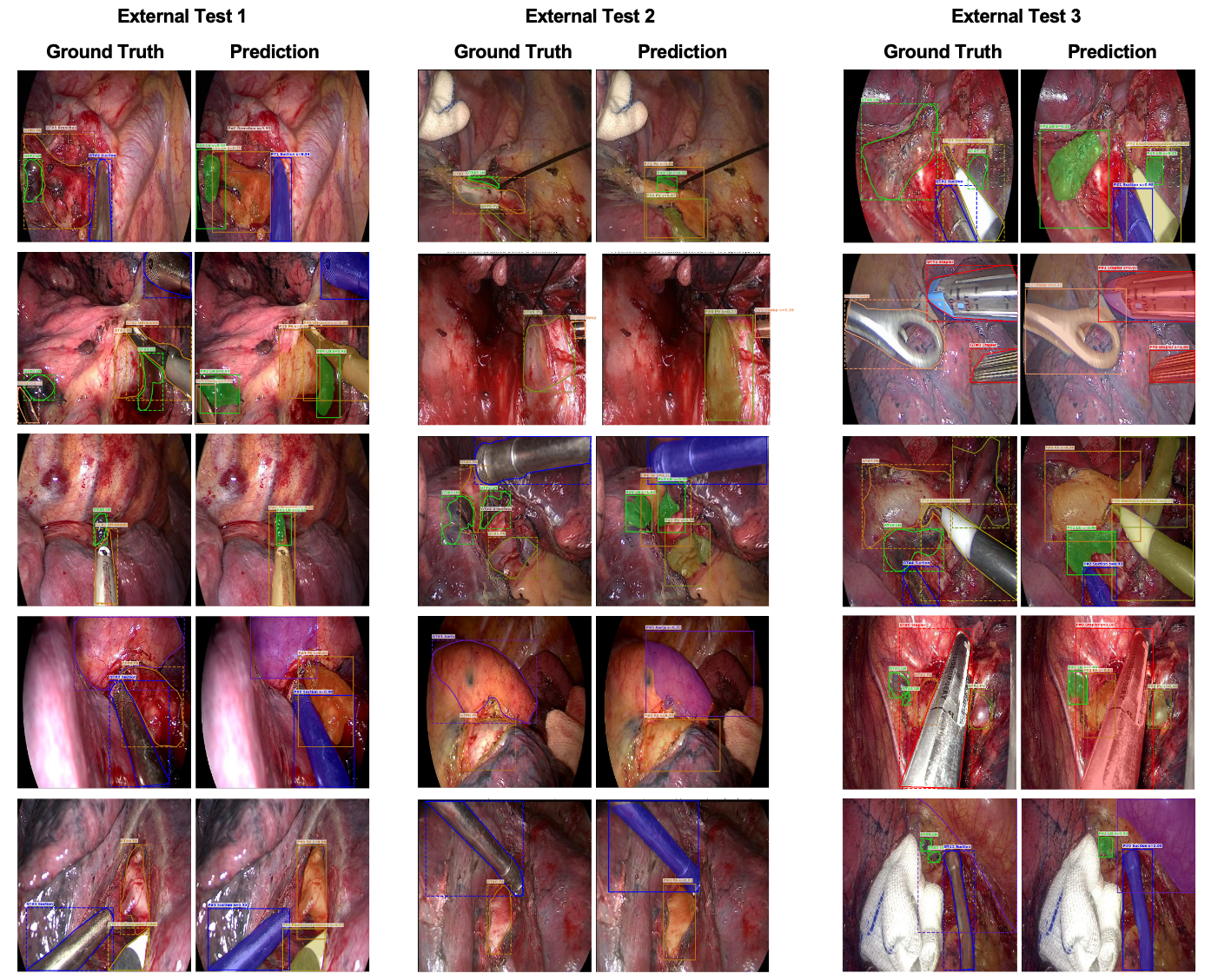
**

**Figure S3** Ablation study on segmentation task

**Figure S4** Image filling task in pre-training

**Figure S5** Examples of incorrect segmentation and classification

**
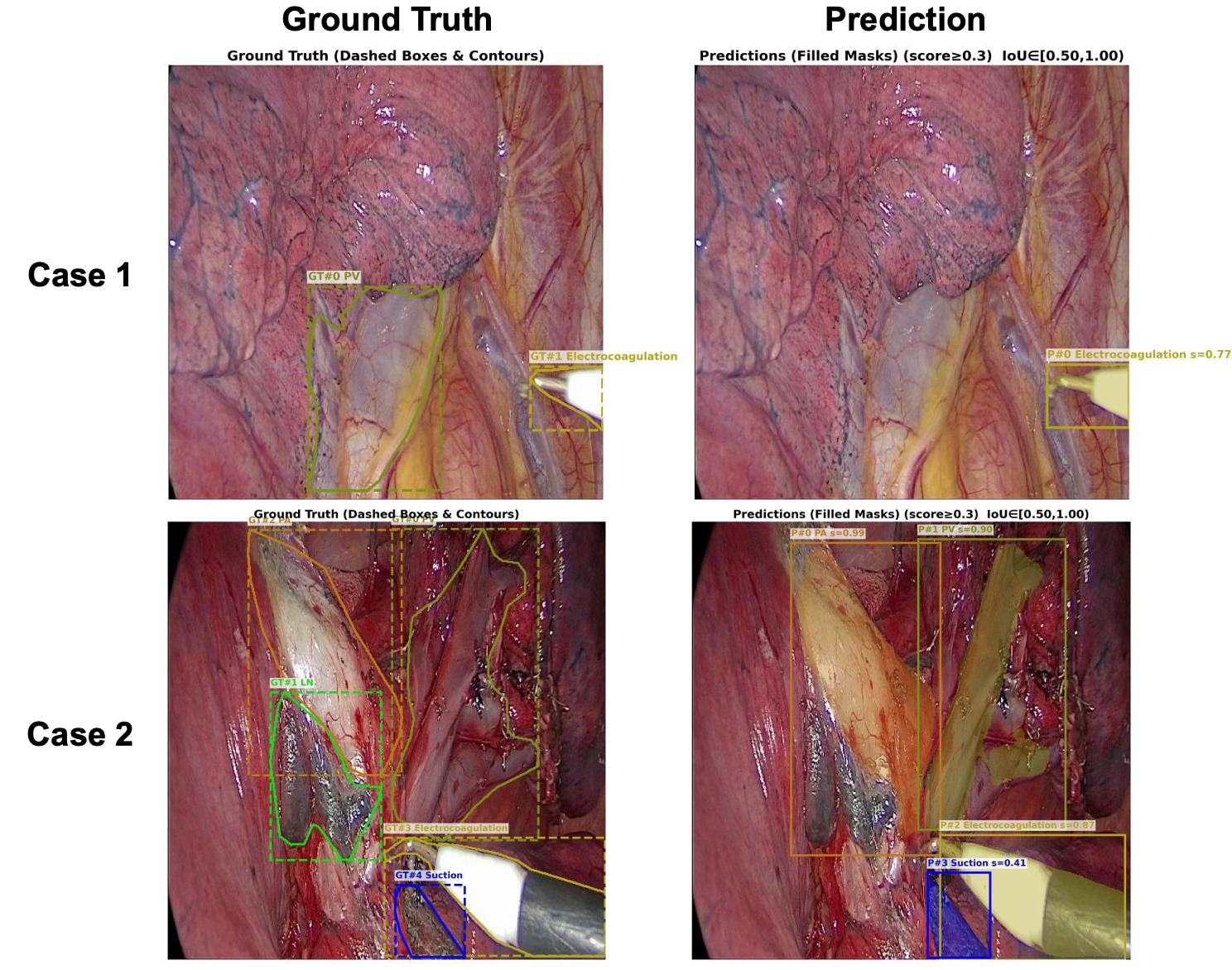
**

Supplementary Figure 5 presents a failure analysis of our model. It highlights two main error types: a missed identification of the superior pulmonary vein (Case 1) and a failure to segment a lymph node (Case 2). These errors likely occurred due to the structures' appearance in atypical contexts. Improving the model's robustness to such uncommon scenarios will be a priority for future research

**Figure S6** Co-occurrence loss of each annotation in the left and the right VATS lobectomy

**Supplementary-**Annotation framework for surgical video

***Initial process of the included videos***

All the surgical videos were cut into one frame per second (1 fps) after frame extraction, generating more than one million frames in total for annotation, and the frames in every video were consecutively numbered for easily record and index. And the processed videos regarding five kinds of VATS lobectomy were 1:1 randomly allocated to thoracic surgeons for AI model-building data annotation.

***Annotation workflow***

Six qualified thoracic surgeons divided into two groups, engaging in the annotation of surgical structures and surgical phase. In general, an annotated video was firstly accomplished by one surgeon and then was reviewed and checked through down by the second and the third annotators. In this process, any intragroup disagreement was properly resolved by discussion. This workflow would ensure an intragroup annotation agreement in full measure. And to assess the impact of annotation subjectivity on data quality, we calculated the Kappa scores between the two groups.

***Segmentation annotation task***

We used the Labelme software (Massachusetts Institute of Technology, Massachusetts, USA) to outlining the shape of the instruments and anatomy in image frame by frame for accumulating segmentation datasets.

The annotation frequency was roughly 1/20 across the whole surgical video. On average, each video included 220 frames of segmentation annotations (ranging from 3.1% to 5.5% per video). According to the diversity and complexity of the scene, the frequency of specific frames contained major step, complicated scene, uncommon and less typical scene was relatively lifted up, to about 1/3 to 1/10, depended upon the surgeons’ subjectivity.

We identified the commonly used instruments and all anatomy involved in VATS lobectomy as annotated structures. To better standardize the surgical structure annotation process, we formulated the following rules.

The principal

- Outline the boundary of objects with accurate points in proper density
- Related objects in one frame of images should be all annotated
- The requency of annotated frames should be high enough for covering all kinds of shape of objects in various major scene
- Emphasize annotation integrity of objects shapes and avoid structural fragmentation
- Do not overlap the annotations

The details

- If the head of instruments were occluded by something, lacking specificity, this atypical feature was more labeled;
- The blurred image caused by camera shaking was appropriately multi-labeled;
- Attention be paid to the interference of electrocoagulation charred tissue, black line head and carbon black on the surface of lung tissue in lymph node labeling;
- Residual threads, sutures, hemoclip, and needles are ignored in the labeling;
- Instruments such as scissors, grasper, and curved forceps are labeled as “clamp”;
- Annotated pulmonary vessels based on the observation of vascular sheath in dissection;
- Delineated objects along the color difference as far as possible;
- When a pulmonary vessel has discernable bifurcation, and the bifurcation boundary is greater than 2/3, it is marked as two objects and otherwise marked as one;
- The Bronchopulmonary vascular stump was marked;
- Anatomic structures would not annotated if they were strongly occluded and fragmented

Figure The interface of the labelme procedure and the surgical structure annotation.


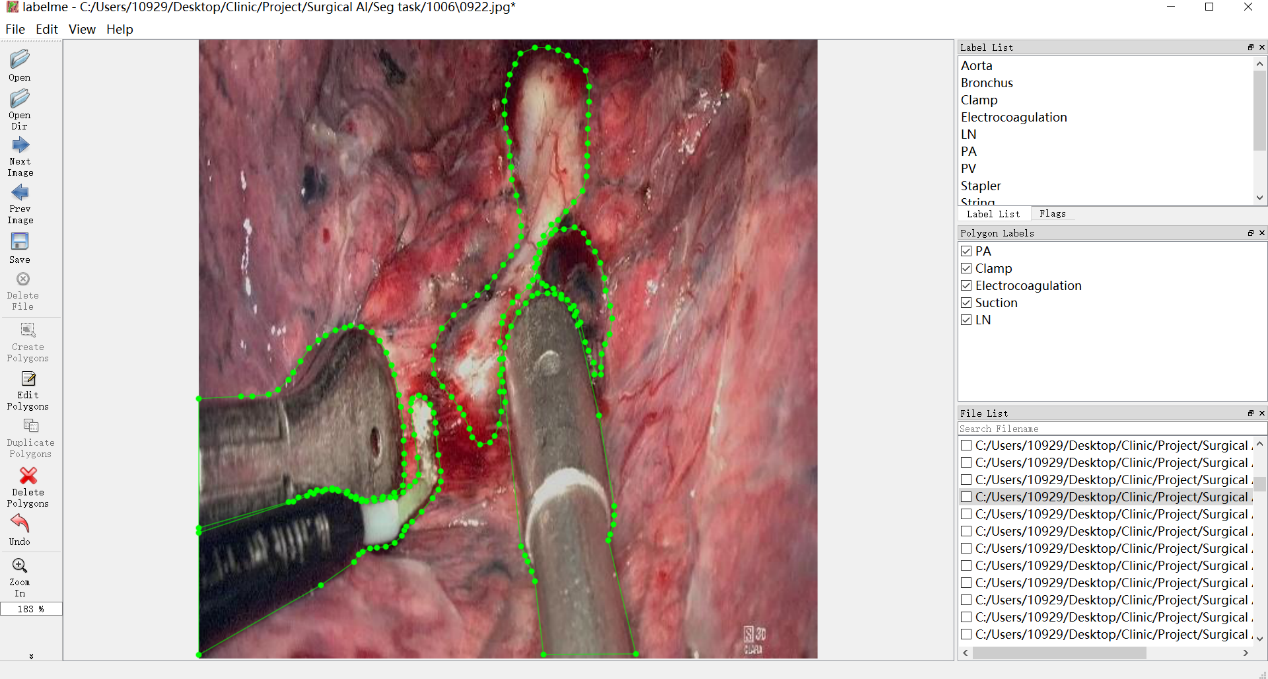


***Classification annotation task***

We used the Excel worksheet to establish a phase classification database by recording the specific surgical phase and the corresponding frame's serial number of the start and end points. The definition of the surgical phase was shown in the *supplementary table 1*. According to the definition, the frame number of the phase start and the endpoint were recorded throughout the whole video, depending upon the surgeons’ perception. More than one million frames were all annotated with surgical phase information.

The surgical phase in this study was the whole description of a specific process without certain details, which were regarded as major phases, for example, the management of pulmonary vessels consists of the dissection, isolation and division action, which were deemed as minor phases. In practice, we further annotated the minor phases on this basis, which entail more effort, and are being explored and analyzed.

The principal

- Do not mainly overlap the phase annotations, with slight overlap permitted
- The cutoff point between two successive phase should be more distinctive.

Figure The demonstration of the surgical phase annotation.


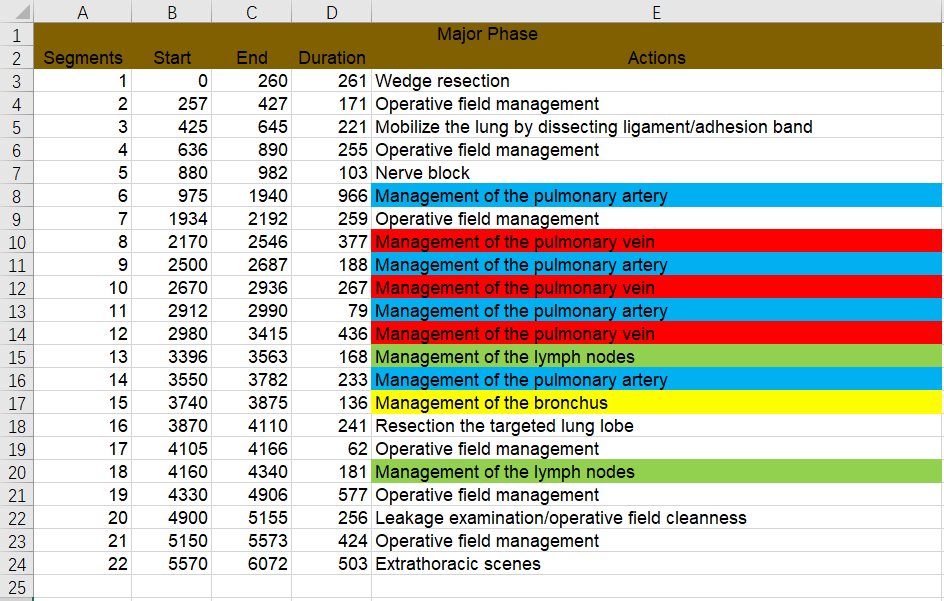

Supplement: Supplementary file 1 — Table S1a: Annotation data characteristics for training set Table S1b: Annotation data characteristics for validation set Table S2 The overall segmentation performance of the LungSurg trained in a single center and validated on the external validation set Table S3: Performance of LungSurg on the External Validation Set, Stratified by Center Table S4: Ablation experiments with the SurgSeg in internal validation Table S5: The overall classification performance of the LungSurg trained in a single center on the external validation set. Table S6: Ablation experiment results of SurgClass (TOP1 and TOP3 ACC for each frame in the internal validation set) Table S7: Definition of each phase of Video‐assisted thoracoscopic (VATS) lobectomy Figure S1: Duration of each surgical phase in the left (A) and right (B) lung Figure S2: Visual assessment of the model's segmentation performance on surgical scenes from external validation centers Figure S3: Ablation study on segmentation task Figure S4: Image filling task in pre‐training Figure S5: Examples of incorrect segmentation and classification Figure S6: Co‐occurrence loss of each annotation in the left and the right VATS lobectomy Supplementary ‐Annotation framework for surgical video [file MCO2-7-e70613-s002.docx]
